# Food Safety Assessment Based on Determination of Lipid Degradation Products in Meat Products

**DOI:** 10.3390/foods15193409

**Published:** 2026-09-24

**Authors:** Jorge Lopez-Tellez, Enoch Olvera-Ureña, Rubén Domínguez-Valencia, Mauricio Opazo-Navarrete, Jose A. Rodriguez

**Affiliations:** 1 Area Academica de Quimica, Universidad Autonoma del Estado de Hidalgo, Carr. Pachuca-Tulancingo Km. 4.5, Mineral de la Reforma 42184, Hidalgo, Mexico; lo319035@uaeh.edu.mx (J.L.-T.); ol278255@uaeh.edu.mx (E.O.-U.); 2 Centro Tecnoloxico da Carne, Avenida de Galicia N° 4, Parque Tecnolóxico de Galicia, San Cibrao das Viñas, 320900 Ourense, Spain; rubendominguez@ceteca.net; 3 Agriaquaculture Nutritional Genomic Center (CGNA), Temuco 4780000, Chile; mauricio.opazo@cgna.cl; 4 Núcleo de Investigación en Producción Alimentaria, Facultad de Recursos Naturales, Universidad Católica de Temuco, Rudecindo Ortega 02950, Temuco 4813302, Chile; 5 FoodTech SpA, Temuco 4780000, Chile

**Keywords:** lipid oxidation, meat, food safety, quality control, aldehydes, oxysterols

## Abstract

Lipid degradation is one of the main factors contributing to the deterioration of meat and meat products, significantly affecting sensory and nutritional properties and leading to the formation of multiple potentially toxic compounds that diminish food quality and negatively impact health. In this regard, given the different oxidation mechanisms and multiple factors that influence them, it is necessary to identify this process to guarantee the quality and safety of meat products. For this purpose, there are international regulations which allow the identification of the oxidation state in meat, however there are no specific regulations for some of these oxidation products. Consequently, due to their relevance, the qualitative and quantitative determination of these compounds represent useful tools to evaluate the oxidative state of meat to guarantee quality and safety of meat products. In this context, this review addresses the fundamentals of lipid oxidation in meat and meat products, the main compounds generated during this process, and the methodologies used for their determination.

## 1. Introduction

Lipids constitute a wide variety of complex biomolecules defined as hydrophobic or amphipathic compounds, including fatty acids, phospholipids, sterols, sphingolipids, and terpenes, among others [1]. They are of great biological importance, as they are responsible for multiple functions in organisms, including roles in cellular structures, metabolic pathways, and thermoregulation processes. Furthermore, lipids serve as the primary source of energy reserves, provide essential fatty acids, and facilitate vitamin absorption; consequently, they are indispensable components of human nutrition and diet [2], although excessive intake of certain lipids as well as some compounds generated from them has been associated with an increased predisposition to developing various diseases [3].

In general, lipids are present in almost all food products, but this review is centered on meat and meat products. These foods have a high lipid content, the quantity and composition of which depend on the animal species including beef, pork, poultry, and fish [4] and are also influenced by age, sex, feeding system, and tissue type [5]. The primary lipids found in these meat sources and products are phospholipids and triglycerides compounds mainly fatty acids. Fatty acids molecules consisting of a long chain of carbon and hydrogen atoms with a carboxylic acid group at one end, are considered the basic structural units of lipids [6]; they are classified as saturated, monounsaturated, or polyunsaturated based on the presence of unsaturation (carbon–carbon double bonds) [7].

Based on their structural composition, lipids can be classified as simple, complex, or derived [8]. Triacylglycerols are the most abundant simple lipids in meat products, phospholipids and glycolipids (complex lipids) are the main sources of polyunsaturated fatty acids [9] while sterols, free fatty acids are the most representative derived lipids [10]. In this regard, the various types of lipids are primarily distributed among subcutaneous, intermuscular, and intramuscular adipose tissues [11].

On the other hand, in meat and meat products, lipids not only contribute significantly to nutritional value but also to sensory properties, as they are precursors to numerous compounds that modify odor, flavor, texture, and appearance [12]. These compounds are generated from lipid degradation and allow for the development of meat’s characteristic aromas. However, uncontrolled degradation due to processing, storage, distribution, and display for sale generates undesirable flavors and odors, as well as nutritional losses and food safety risks [13]. Lipid degradation can involve hydrolysis or direct oxidation. Lipid oxidation in meat occurs via autoxidation, photo-oxidation, and enzymatic oxidation mechanisms [14] and is influenced by various factors, including fatty acid composition, oxygen availability, temperature, light exposure, and the presence of pro-oxidant compounds [15,16].

Autoxidation is generally the most significant mechanism and comprises three fundamental stages: initiation, propagation, and termination [16]. During these stages, lipids undergo a series of chemical reactions resulting in the formation of primary oxidation products mainly lipid hydroperoxides which subsequently decompose into a wide range of secondary and tertiary products. These include aldehydes, ketones, alcohols, hydrocarbons, esters, acids, epoxides, hydroxylated compounds, oxysterols, oligomers, and polymers [17,18,19]. Furthermore, certain lipid oxidation products have been shown to have potentially toxic properties when they react with other biomolecules, interfering with the organism and causing mutagenic, genotoxic, cytotoxic, and carcinogenic effects. Therefore, their formation represents a public health concern [5].

Although ingredients such as salt, nitrites, spices, and antioxidants as well as heat treatments are added to meat and meat products to inhibit lipid oxidation [20,21], this process remains a significant issue due to the variety of lipids (such as triacylglycerols, sterols, phospholipids, and polyunsaturated fatty acids) that are susceptible to oxidation [5,22]. Consequently, determining lipid oxidation products is a crucial tool for assessing the quality, oxidative stability, and shelf life of meat and meat products. Various approaches have been developed to monitor lipid oxidation by measuring lipid hydroperoxides, conjugated dienes, and other secondary oxidation compounds; furthermore, instrumental techniques such as UV-Vis and fluorescents spectrophotometry, gas chromatography (GC), high-performance liquid chromatography (HPLC), and mass spectrometry offer greater specificity for individual oxidation products [23].

In this regard, this review aims to identify the primary lipid oxidation products generated in meat and meat products and to analyze the most used methodologies for their qualitative and quantitative determination. It also addresses the potential application of these products as indicators of oxidative deterioration, quality, shelf life, and safety, highlighting their relevance for monitoring the stability and quality of meat and meat products.

## 2. Classification of Lipids

They can be classified according to their physical characteristics, polarity, nutritional value, and structural composition; the latter is the most used method, categorizing them as simple, complex, or derived lipids [8]. Simple lipids are fatty acid esters comprising acylglycerols and waxes; they generally constitute the largest proportion of total lipids in meat [24]. Acylglycerols (Figure 1a) are the primary simple lipids; they are formed by linking long-chain fatty acids to glycerol via ester bonds [25], resulting in monoacylglycerols, diacylglycerols, or triacylglycerols [26] that contain saturated, unsaturated, and polyunsaturated fatty acids. Waxes are esters composed of fatty acids and long-chain monohydric alcohols containing between 8 and 36 carbon atoms [10]. The main fatty acids (Figure 1b) identified in beef, pork, poultry, and fish are: myristic acid, palmitic acid, stearic acid, oleic acid, erucic acid, eicosenoic acid, linoleic acid, α-linolenic acid, arachidonic acid, eicosapentaenoic acid, docosahexaenoic acid, and docosapentaenoic acid [27]; however, their proportions depend on the animal’s diet as well as the specific tissue [28].

Complex lipids are important components of meat tissues; they contain elements such as phosphorus, nitrogen, or sulfur, with phospholipids and sphingolipids being the primary types [29]. These constitute essential components of meat cell membranes [1,30] and represent the main source of various polyunsaturated fatty acids [9]. Phospholipids make up the principal group of complex lipids and are considered the most important class of polar lipids; they possess amphiphilic properties due to the presence of a hydrophobic region formed by fatty acid chains and an attached phosphate group that confers on a polar character [31]. Phospholipids can be classified as glycerophospholipids and sphingophospholipids. Glycerophospholipids are composed of a glycerol molecule linked to two acyl chains and a functionalized phosphate group [32]. Predominant glycerophospholipids include phosphatidylcholine, phosphatidylethanolamine, phosphatidylserine, phosphatidylinositol, phosphatidylglycerol, cardiolipin, and phosphatidic acid; these compounds have been described primarily in muscle tissue [29,33]. Sphingophospholipids, meanwhile, are compounds consisting of a sphingosine molecule linked via an amide bond to a fatty acid (Figure 2). The most abundant sphingophospholipid in animal tissues is sphingomyelin, which consists of a ceramide backbone linked to a phosphorylcholine or phosphorylethanolamine head group [32].

On the other hand, sphingolipids constitute another of the main families of complex lipids, characterized by having a long chain sphingoid base through the union of a fatty acid through an amide bond. In this regard, ceramide formed by the attachment of a fatty acid to the sphingoid base serves as the central structural intermediate from which more complex sphingolipids, such as sphingomyelins, glycosphingolipids, sulfatides, and lysosphingolipids, are generated (Figure 3) [34,35,36].

In meat, these membrane-associated lipids are present in relatively smaller quantities than simple lipids, but their structural and metabolic functions make them relevant to the composition and function of muscle tissue.

Derived lipids are molecules synthesized through the modification of simple and complex lipids, and they include steroids and terpenoids [26]. Furthermore, compared to acylglycerols and sphingolipids, they are found in relatively low concentrations in meat and meat products. Steroids naturally present in muscle and adipose tissues [37,38], include sterols and sterol esters; they are organic molecules distinct from other lipids. Their basic structure consists of four rings (three cyclohexane rings and one cyclopentane ring) derived from cyclopentanoperhydrophenanthrene (Figure 4a) [26]. Cholesterol (Figure 4b) is the predominant steroid in animal tissues [37]. In addition to its structural function, it is an essential component for the synthesis of steroid hormones, bile acids, and vitamin D [38]. On the other hand, terpenoids constitute a diverse group of organic compounds derived from isoprene, a five-carbon hydrocarbon. Terpenoids form a broad and structurally diverse group of compounds derived from five-carbon isoprene units (Figure 4c) and include certain fat-soluble vitamins, carotenoids, and some coenzymes [29].

## 3. Lipid Composition in Meat

The primary lipids making up meat tissue are simple lipids (acylglycerols) and complex lipids (phospholipids), while lipids derived such as free fatty acids, terpenoids, and cholesterol contribute to a lesser extent. These compounds are located and distributed across various fat depots, including subcutaneous, intermuscular, and intramuscular adipose tissues. Phospholipids are key components of cell membranes and contain a high proportion of polyunsaturated fatty acids, whereas triglycerides serve as the primary energy reserve [39,40]. However, there is significant variation depending on factors such as muscle type, breed, age, sex, diet, and production system. Lipid composition is a major determinant of nutritional value, sensory characteristics, and susceptibility to lipid oxidation following slaughter [28].

Although the largest quantity of lipids is found in the acylglycerol fraction, phospholipids have been identified as playing a fundamental role in the development of lipid oxidation. This is due to their high reactivity stemming from the arrangement of lipids within membranes, which facilitates the propagation of oxidation [41] and their higher content of polyunsaturated fatty acids compared to triglycerides, making them more susceptible to oxidation [5].

Intramuscular fat is located between muscle fibers and significantly influences sensory characteristics such as juiciness, tenderness, and flavor. Generally, chicken meat contains the lowest levels of intramuscular lipids, whereas pork, beef, and lamb typically exhibit higher concentrations [42]. Furthermore, fatty acid composition is an important consideration. Meat from ruminants (beef and sheep) contains a higher proportion of saturated and monounsaturated fatty acids; notable examples include palmitic and stearic acids (saturated) and oleic acid (unsaturated). On the other hand, monogastric species (such as pigs and poultry) typically exhibit a higher proportion of polyunsaturated fatty acids [43]. These differences are linked to the metabolism of each species and particularly in ruminants to ruminal biohydrogenation, a process in which microorganisms modify dietary unsaturated fatty acids before they are incorporated into the animal’s tissues [44]. The proportion of these fatty acids can vary depending on the animal’s diet and intramuscular fat content. The primary fatty acids found in pork and chicken meat are linoleic acid and α-linolenic acid; these are essential for metabolism but cannot be synthesized by the organism (their main source is dietary). In pigs and poultry, these two fatty acids are metabolized into long-chain derivatives such as arachidonic acid, eicosapentaenoic acid, docosapentaenoic acid, and docosahexaenoic acid [45].

In this regard, susceptibility to oxidation has been described as proportional to the number of degrees of unsaturation present in fatty acids; thus, the greater the number of double bonds, the higher the susceptibility to oxidation [46]. This is particularly significant in poultry meat, given its higher degree of unsaturation compared to meat from other species [47]. Furthermore, trans isomers have been shown to exhibit greater stability than cis isomers. Susceptibility also depends on fatty acid chain length, with long-chain fatty acids being more susceptible [5].

## 4. Lipid Oxidation Mechanism in Meat

Meat and meat products contain all three types of lipids; therefore, they are susceptible to lipid degradation which generates a wide variety of degradation products. Lipid degradation involves two main processes: (1) lipid hydrolysis which breaks the ester bond, allowing for the release of fatty acids and other compounds derived from lipids found in acylglycerols, waxes, and phospholipids and (2) lipid oxidation, the stage in which fatty acids begin to degrade and form multiple compounds. However, lipid hydrolysis is not a prerequisite for oxidation, as fatty acids esterified within intact molecules can also undergo oxidation [13]. This is particularly relevant for tissue sphingolipids, which contain high proportions of polyunsaturated fatty acids. Figure 5 presents the three pathways describing the oxidation process: autoxidation, photo-oxidation, and enzyme-catalyzed oxidation [14]. Lipid oxidation significantly impacts food quality and safety due to its contribution to the development of undesirable odors and flavors and the formation of potentially toxic oxidation products [13].

### 4.1. Lipid Autooxidation

Autooxidation is the most significant lipid oxidation process contributing to oxidative deterioration in meat and meat products. It is a continuous, free-radical-mediated reaction that generally originates predominantly in the unsaturated and polyunsaturated fatty acids found in sphingolipids, phospholipids, waxes, and triacylglycerols, leading to the formation of oxidative deterioration products [48].

This type of oxidation is described in terms of three distinct stages: initiation, propagation, and termination. During the initiation stage, a hydrogen atom is abstracted from an unsaturated or polyunsaturated fatty acid (specifically from the bis-allylic position) [49]. In meat, this stage is promoted by the presence of metals, proteins, reactive oxygen species, and other pro-oxidant conditions arising from animal slaughter [49]. The resulting lipid radical reacts rapidly with molecular oxygen to form a lipid peroxyl radical [15]. The presence of natural antioxidants delays the initiation stage and promotes a slower rate of radical formation [13].

Subsequently, during the propagation stage, lipid peroxyl radicals abstract hydrogen atoms from other fatty acids, producing lipid hydroperoxides and regenerating lipid radicals. The generated lipid radicals react with molecular oxygen, sustaining the chain reaction. Lipid hydroperoxides are the primary products of lipid oxidation and can accumulate during the early stages of oxidation in meat [50]. However, they are relatively unstable and can decompose, particularly under heat and other conditions characteristic of meat systems.

Their decomposition yields alkoxyl, peroxyl, and hydroxyl radicals, among other reactive intermediates that undergo further reactions such as β-scission and hydrogen abstraction generating secondary oxidation products like aldehydes, ketones, alcohols, hydrocarbons, and other carbonyl compounds [51,52]. Propagation is the stage that significantly accelerates the rate of lipid oxidation [53]. Finally, the termination stage involves reactions that eliminate or reduce the chain-propagating radicals; these include radical-radical recombination, disproportionation, and reactions in which antioxidant molecules donate hydrogen atoms or electrons to lipid radicals. This leads to the formation of stable or low-reactivity compounds [26]. The numerous by-products resulting from the entire oxidation process are responsible for the deterioration of the sensory and nutritional qualities of meat and meat products [15]. Although the free-radical autoxidation mechanism is the most widely cited and explains various formation processes, it does not provide a detailed and comprehensive description of all the resulting products [26].

### 4.2. Lipid Photooxidation

Lipid photooxidation contributes substantially to the oxidative deterioration of meat and meat products during processing, storage, and retail display. Unlike classical free-radical autoxidation, photooxidation depends on light, oxygen, and a photosensitizing molecule. For this oxidation mechanism to occur, photosensitizers present in the meat must first absorb and transition to an electronically excited state, enabling them to participate in energy-transfer reactions [54]. This type of oxidation can occur via two mechanisms. The Type I mechanism occurs when the excited photosensitizer participates in electron or hydrogen transfer reactions with surrounding molecules, generating radical species that subsequently react with oxygen. Depending on the sensitizer and environmental conditions, superoxide anions and other reactive oxygen species are generated [55], while Type II mechanism, the excited photosensitizer transfers energy to molecular oxygen, produces singlet oxygen; this reacts directly with fatty acids to yield lipid hydroperoxides through reactions that do not require the initial formation of lipid alkyl radicals [56].

Furthermore, pigment proteins in meat influence lipid photo-oxidation by interacting with light and participating in photochemical processes. Among these proteins is myoglobin, which acts as a photosensitizer due to its heme/protoporphyrin structure and is primarily associated with the formation of singlet oxygen [57]. Similarly, hemoglobin and other heme-containing components can influence oxidative processes by promoting lipid oxidation through various mechanisms involving their redox chemistry, iron-mediated reactions, and interactions with lipid hydroperoxides [54].

However, in meat, photooxidation and autoxidation occur simultaneously; the light-induced formation of singlet oxygen, reactive oxygen species, or lipid hydroperoxides can subsequently fuel free radical oxidation pathways. Consequently, exposure to light can accelerate lipid deterioration through interconnected photosensitization and free radical reactions [15].

### 4.3. Enzymatic Lipid Oxidation

Enzymatic lipid oxidation in meat encompasses a group of reactions in which enzymes contribute to the formation of lipid hydroperoxides and modify the environment of reactive oxygen species and peroxides within muscle tissue. In meat, the contribution of each enzyme depends on its abundance, localization, substrate availability, post-mortem changes, processing conditions, pH, temperature, and enzymatic stability. Consequently, enzymatic lipid oxidation is considered a pathway that interacts with non-enzymatic lipid oxidation [15].

Lipoxygenase is a fundamental enzyme involved in enzymatic oxidation, because it directly catalyzes the oxygenation of polyunsaturated fatty acids that contain a “cis” system. Lipoxygenase incorporates molecular oxygen into susceptible fatty acid substrates (generally linoleic and linolenic acids), producing specific lipid hydroperoxides. The reaction involves the formation of a hydroperoxide with characteristic positional and stereochemical configurations through the abstraction of a hydrogen atom from the fatty acid, followed by the addition of oxygen. The generated hydroperoxides may subsequently undergo enzymatic or non-enzymatic decomposition to form secondary oxidation products [58].

Oxidoreductases are another class of enzymes that influence lipid oxidation by regulating the availability of reactive oxygen species or by modifying lipid hydroperoxides. In this regard, oxidoreductases such as xanthine oxidase and components of cytochrome systems do not directly oxidize lipids; rather, they modulate reactive oxygen species, which can subsequently participate in non-enzymatic lipid oxidation [15].

In contrast, peroxidases and other related peroxide-metabolizing enzymes influence lipid oxidation through their interaction with hydrogen peroxide and lipid hydroperoxides. Depending on the specific enzyme, they may promote the formation of reactive intermediates or contribute to the elimination of peroxides [5].

Cyclooxygenase is another enzyme associated with lipid oxidation; it is an oxygen-dependent enzyme capable of oxygenating fatty acids and forming hydroperoxide containing intermediates. However, its physiological function is primarily linked to eicosanoid biosynthesis rather than lipid oxidation; consequently, its contribution to oxidative deterioration should not be regarded as a major general pathway of lipid degradation [58].

## 5. Relevance of Lipid Oxidation Products to Meat Quality

Lipid oxidation in meat generates multiple primary and secondary oxidation products, the formation of which is influenced by fatty acid composition. In particular, the higher the degree of fatty acid unsaturation, the greater the susceptibility of lipids to oxidative degradation; consequently, polyunsaturated fatty acids are prone to oxidation [5,59,60].

Hydroxyoctadecadienoic acids (HODEs) are particularly significant as stable oxygenated derivatives of linoleic acid. Two main regioisomers stand out 9-hydroxy-octadecadienoic acid (9-HODE) and 13-hydroxy-octadecadienoic acid (13-HODE) which originate from their corresponding hydroperoxides (9-and 13-hydroperoxyoctadecadienoic acids) formed during oxidation [61]. HODE formation can occur via both enzymatic and non-enzymatic pathways. Lipoxygenases catalyzed the regio- and stereospecific formation of linoleic acid hydroperoxides, whereas free radical-mediated oxidation generates mixtures of positional isomers and stereoisomers [62]. The formation of *trans-trans* HODE isomers have been proposed as a more specific indicator of free radical-mediated lipid peroxidation, given that *cis-trans* HODEs can arise from both enzymatic and non-enzymatic pathways [61]. Consequently, HODE formation provides insight into degradation mechanisms by revealing the oxidative pathway involved in meat and meat products. Therefore, they should be interpreted differently from other degradation compounds; as relatively stable lipid oxidation products, they serve to complement other techniques used for analyzing lipid oxidation [63].

HODE levels have been associated with the progression of certain diseases. Higher HODE levels were found in atherosclerotic lesions. Furthermore, 13-HODE has been linked to the development of prostate cancer [64], breast cancer [65] and colon cancer [66]. Despite their importance, no maximum level has been established for meat and meat products [67], currently, only an acceptable level of lipid hydroperoxides of 5–10 mmol kg^−1^ has been established for oil or fat [68].

On the other hand, the breakdown of primary products mainly lipid hydroperoxides generates numerous secondary compounds, including aldehydes, ketones, alcohols, hydrocarbons, and oxygenated lipid derivatives [5]. Among these, aldehydes are particularly notable due to their high reactivity with biomolecules and their contribution to the sensory properties of meat [69,70]. Aldehydes generated during lipid oxidation include aliphatic, α,β-unsaturated, and oxygenated aldehydes [70]. Some of these compounds have demonstrated mutagenic [71], genotoxic [72], and carcinogenic effects [69,73]. Currently, some of the aldehydes considered of priority interest listed by the IARC (International Agency for Research on Cancer) by the World Health Organization include formaldehyde and acetaldehyde in group 1 (carcinogenic), acrolein and crotonaldehyde in groups 2A and 2B respectively (probable and possible carcinogens); Glyoxal, methylglyoxal, glutaraldehyde, and malondialdehyde are in group 3 (there is insufficient evidence) although, their mutagenic properties and high reactivity have been demonstrated [73]. However, these hazard classifications and toxicological findings should not be interpreted as evidence of food safety risk; although their presence in meat and links to certain diseases have been documented, dietary exposure limits have not been established for many of these hazardous compounds [74].

Long chain aldehydes including pentanal, hexanal, heptanal, octanal, nonanal, and decanal are important products of lipid oxidation. Hexanal and nonanal are commonly associated with omega-6 oxidation [75], whereas propanal is primarily generated from omega-3 [76]. These aldehydes are frequently used as indicators of lipid oxidation and contribute to the characteristic aroma of meat [5]. Other aldehydes of interest, such as malondialdehyde, can be generated from the oxidation of various fatty acids, while 4-hydroxy-2-nonenal is predominantly associated with omega-6 oxidation, particularly that of linoleic and arachidonic acids. 4-hydroxy-2-hexenal is primarily derived from the oxidation of omega-3, including α-linolenic and docosahexaenoic acids [77,78]. Furthermore, these aldehydes have been reported to react with amino acid residues in proteins, potentially altering their structure and function [78]. In meat, membrane phospholipids serve as important substrates for the formation of these secondary oxidation products [79].

Accordingly, other secondary products specifically noted to affect sensory quality include ketones such as 2-heptanone, 2-octanone, 2-nonanone, and 3-octanone [80] as well as alcohols (pentanol, hexanol, octanol, and 1-octen-3-ol) and hydrocarbons (pentane and hexane) [5].

Phospholipid oxidation is particularly significant in cooked, refrigerated meat. This process is strongly linked to the development of warmed-over flavor (WOF) a characteristic rancid, cardboard-like, astringent, or metallic flavor that emerges when cooked meat is refrigerated and subsequently reheated [81]. WOF is primarily associated with the oxidation of membrane phospholipids, the degradation of carbohydrates and proteins also contributes to the process, leading to the formation of various volatile organic compounds, including aldehydes, alcohols, ketones, esters, hydrocarbons, and other compounds. The susceptibility of phospholipids to oxidation, combined with the release of pro-oxidant iron during cooking, contributes to the rapid development of WOF during refrigerated storage [82]. Therefore, phospholipid oxidation represents a key link between lipid degradation, sensory deterioration, and the shelf life of cooked meat products; from this perspective, WOF is considered primarily an issue of quality and consumer acceptability [81].

Furthermore, sterols are susceptible to oxidation by reactive oxygen species, leading to the formation of cholesterol oxidation products, or oxysterols [83]. The formation of mono- or polyoxygenated compounds has been described [84], including 20α-hydroxycholesterol, 25-hydroxycholesterol, α- and β-epoxycholesterol, 7α- and 7β-hydroxycholesterol, and 7-ketocholesterol [85,86]. Notably, 7-ketocholesterol is a major cholesterol oxidation product (COP) and can serve as an indicator of cholesterol oxidation [5,86]. Oxysterols have demonstrated mutagenic, angiotoxic, and cytotoxic properties, in addition to contributing to the development of atherosclerosis and certain types of cancer [87].

## 6. Regulatory Standards Applicable to Lipid Oxidation Products

Despite the important role of numerous compounds generated by lipid oxidation in meat and meat products, there is currently no official analytical methodology, either quantitative or qualitative, for determining any of these compounds. However, international standards issued by the International Organization for Standardization (ISO) can be used to analyze groups of lipid oxidation compounds in meat and meat products. Two standards are applicable: ISO 3960:2017 is the official method for determining the peroxide value in the meat industry for products such as sausages, cured meat, and processed meats. The peroxide value is the most widely used indicator for assessing primary lipid oxidation. It quantifies the hydroperoxides formed in the early stages of the oxidative process by iodometric titration. The method is based on the reduction in hydroperoxides by the iodide ion, releasing iodine, which is subsequently determined by titration with sodium thiosulfate through the formation of the triiodide ion [88]. Although widely used due to its simplicity and low cost, this method has limitations caused by instability of hydroperoxides, which is affected by the presence of oxygen, reaction temperature, and other interfering substances present in the meat [17]. Similarly, Association of Official Agricultural Chemists (AOAC) has developed several methodologies that complement the procedures used for food quality control. In the case of meat products, AOAC 965.33.12 methodology can be used to determine peroxide value, analogous to ISO 3960:2017. This method is based on the reaction of lipid hydroperoxides with iodide ion, which generates molecular iodine, the quantity of which is determined by titration with sodium thiosulfate [89].

The most recent standard used is ISO 6885:2016, which establishes the technique for determining the *p*-anisidine index. The method is based primarily on the determination of unsaturated aldehydes through the reaction of the amino group contained in *p*-anisidine with the carbonyl group of the aldehyde, forming a Schiff base. The resulting compound is determined by ultraviolet-visible spectroscopy at 350 nm [90]. However, given the complexity of meat samples, lipid extraction is necessary before analysis [91].

On the other hand, some AOAC methodologies were not specifically developed for the determination of oxidation products, however, they can be used as complementary tools to indirectly assess the degree of oxidation that lipids can undergo. Among these is the determination of the iodine value (AOAC 993.20), which allows for the estimation of the degree of unsaturation of lipids [92]. The method is based on the halogenation of double bonds by adding an excess of iodine monochloride, the unreacted iodine is reduced to molecular iodine, which can then be determined by titration with sodium thiosulfate. In this way, it is possible to estimate the double bonds susceptible to oxidative processes.

Furthermore, using the AOAC 957.13 methodology, it is possible to indirectly assess lipid oxidation, as it is based on the spectrophotometric determination of polyunsaturated fatty acids and conjugated dienes. Conjugated double bond systems absorb in the ultraviolet region, approximately between 233 and 271 nm, wavelengths primarily associated with conjugated diene and triene structures, respectively. For their determination, lipids are dissolved in isooctane, and the concentration of these compounds is calculated using the Lambert-Beer law. The formation of conjugated dienes and trienes can be associated with the initial stages of polyunsaturated fatty acid oxidation; therefore, this methodology can be complementary in providing an approximation of the oxidation processes in lipids present in meat products [93].

## 7. Methods for Determining Lipid Oxidation Products

Because of the toxicological properties of lipid oxidation products and their significant impact on the quality and safety of meat products, analytical methodologies have been developed for their qualitative and quantitative determination. These methods enable the detection of specific compounds or groups of oxidation products and must offer adequate accuracy, precision, and sensitivity for reliable assessment. In general, methods for evaluating lipid oxidation can be classified as direct or indirect. Direct methods involve the detection and quantification of specific compounds generated during lipid oxidation, whereas indirect methods assess the consequences of oxidation through changes in sensory characteristics or other secondary responses [94]. Consequently, direct methods provide more specific information regarding the extent of lipid oxidation and the compounds formed, making them particularly valuable for assessing both meat quality and the potential presence of toxic oxidation products.

### 7.1. Methods for the Determination of Primary Lipid Oxidation Products

Lipid hydroperoxides are primary oxidation products typically determined using volumetric methods, spectroscopic techniques, chromatographic techniques [95]. The determination of lipid hydroperoxide production is known as the peroxide value. This value is measured via iodometric titration (Figure 6a), as described in official methods (ISO 3960:2017 and AOAC 965.33). The method is defined as the amount of active oxygen (meq or mmol) per kilogram of lipids capable of oxidizing potassium iodide. The peroxide value is a traditional indicator of primary oxidation in meat and meat products. However, it should be noted that hydroperoxides are transient products; consequently, their rates of formation and decomposition can alter the measured value as oxidation progresses, given that they begin to decompose rapidly, potentially leading to a decrease in their concentration. Thus, a low peroxide value may indicate either a sample with minimal oxidation or one that has reached an advanced stage of oxidation [96]. This limitation is particularly relevant for meat products subjected to prolonged storage, cooking, drying, or treatments that promote oxidation. Furthermore, this method has other limitations, as it is influenced by temperature, reaction time, the structure and reactivity of the peroxides, and the presence of oxygen, as well as the fact that the released iodine is absorbed at the sites of fatty acid unsaturation [17].

An alternative to conventional iodometric titration is determination via UV-Vis spectrophotometry. Since lipid hydroperoxides react quantitatively with potassium iodide under acidic conditions to release iodine, the iodine can react with starch to form a blue complex that is measured at 550 nm. Furthermore, in the presence of excess iodide, the released iodine forms the triiodide anion, the concentration of which can be measured at 353 nm [97]. This modification reduces the likelihood of iodine reacting with the highly unsaturated lipids present in meat, which could lead to erroneous results. Applying the iodometric method to meat matrices generally requires prior lipid extraction from the sample. Additionally, experimental conditions must be carefully controlled to ensure high sensitivity [17].

On the other hand, ferrous oxidation has been proposed as an alternative to iodometry for determining hydroperoxides, as it is a simpler procedure and less susceptible to certain interferences associated with atmospheric oxygen. In this method, hydroperoxides oxidize Fe(II) to Fe(III) under acidic conditions (Figure 6b). Subsequently, the ferric ion reacts with a chromogenic compound such as thiocyanate (Figure 6c) or xylenol orange (Figure 6d) to form colored complexes whose absorbance can be measured using UV-Vis spectrophotometry [95]. The ferric thiocyanate method relies on the formation of a red complex between ferric ions and potassium thiocyanate, which exhibits an absorbance maximum near 500 nm. However, values obtained using this procedure have been observed to be 1.5 to 2 times higher than those determined by iodometry. Moreover, it requires a significant volume of solvent and has a more limited peroxide detection interval compared to other ferrous oxidation methods. The method based on ferrous-xylenol orange oxidation relies on a complex formed between ferric ions and xylenol, exhibiting maximum absorbance in the 550–600 nm interval [68]. Furthermore, results obtained using this method showed good correlation with those from the iodometric method. Methods based on ferrous oxidation are notable for their high sensitivity surpassing that of the iodometric method in the determination of hydroperoxides [98]. However, they present certain limitations that must be considered when analyzing meat and meat products. These include a relatively limited linear range and reproducibility that can be affected by matrix characteristics, lipid extraction or purification procedures, the presence of oxidizing or reducing substances, and metal contamination; additionally, certain pigments present in meat matrices can interfere with spectrophotometric measurements [99].

In addition to UV-Vis spectroscopic methods, a method based on fluorescence spectroscopy has been developed for the determination of lipid hydroperoxides; this method offers greater sensitivity and selectivity. The methodology relies on the use of diphenyl-pyrenylphosphine; although this compound is not inherently fluorescent, it reacts upon oxidation by lipid hydroperoxides present in the meat to form a highly fluorescent product (Figure 6e). The intensity of the generated fluorescence is proportional to the amount of hydroperoxides present, allowing for their determination with high sensitivity. The signal is measured using a fluorometer at an excitation wavelength of 351 nm and an emission wavelength of 380 nm [100].

A significant advantage of this methodology for the analysis of meat and meat products is its high sensitivity, as it enables the detection of relatively low levels of hydroperoxides. A particularly relevant advantage of this methodology for the analysis of meat and meat products is that it allows for the determination of total hydroperoxides directly in meat product homogenates, without requiring a prior lipid extraction step. This can simplify the analytical procedure and reduce potential losses or alterations of the hydroperoxides during sample preparation [101].

The formation of conjugated dienes and trienes is one of the methods used to assess the initial stages of lipid oxidation in meat and meat products. During the oxidation of polyunsaturated fatty acids, a rearrangement of double bonds occurs, leading to an increase in conjugated dienes and trienes; these serve as indicators of the early development of lipid oxidation in meat [16]. The determination of conjugated dienes and trienes is performed using UV-Vis spectroscopy. Conjugated dienes exhibit an absorption maximum near 234 nm, whereas conjugated trienes absorb at approximately 268 nm. For analysis in meat, lipids are first extracted using organic solvents such as hexane/isopropanol or chloroform/methanol mixtures and absorbance is subsequently recorded at the corresponding wavelengths [94]. This procedure is relatively rapid and simple, requiring no additional chemical reactions, making it a practical method for monitoring lipid oxidation in meat and meat products. However, the utility of conjugated dienes and trienes is primarily associated with the early phases of oxidation. As the oxidative process progresses, hydroperoxides may degrade, and conjugated compounds no longer accurately reflect the degree of oxidation. Furthermore, this method offers lower sensitivity and specificity compared to other indicators, as certain compounds present in the meat absorb in the same region and interfere with the determination [102].

Therefore, the determination of conjugated dienes and trienes is a useful tool for identifying and monitoring the initial stages of lipid oxidation in meat; however, results must be interpreted with consideration of the meat matrix characteristics and potential spectral interferences. To obtain a more comprehensive assessment of oxidative deterioration, this determination is often conducted alongside other indicators [17].

In addition, liquid chromatography-based methods specifically HPLC have been developed to determine hydroxyoctadecadienoates, enabling the assessment of primary degradation products associated with linoleic acid in meat and meat products. For analysis, lipids are typically extracted from the sample using solid-phase extraction cartridges, followed by separation via HPLC coupled with mass spectrometry [61].

This procedure allows for a more specific determination of linoleic acid-derived oxidation products compared to the overall measurement of other oxidation indicators. Various methods have been developed in this regard; for instance, 13-hydroxyoctadecadienoic acid, 9,10-dihydroxyoctadecenoic acid, and 9,10,13-trihydroxyoctadecenoic acid have been determined specifically and simultaneously in various cured meat products [103], and these same acids along with 9-hydroxyoctadecadienoic acid have been determined in sausages [61]. The direct determination of these compounds allows for more precise monitoring of the formation of primary degradation products and changes in their relative proportions [103].

### 7.2. Methods for the Determination of Secondary Lipid Oxidation Products

During the oxidation process, a greater number of secondary products are generated. Numerous methods exist for determining these products, as they are the compounds associated with adverse health effects, as well as being responsible for unpleasant flavors and odors and the loss of nutritional value. Malondialdehyde is the most widely used marker of secondary lipid oxidation, its determination is commonly based on its reaction with 2-thiobarbituric acid (TBA) under acidic conditions and at high temperatures (90–100 °C) [94]. This reaction generates a compound that is detected by spectrophotometry [104]. Following the reaction, the sample undergoes a treatment that commonly involves the use of trichloroacetic acid to precipitate proteins and remove matrix components that cause interference. However, the TBA assay lacks specificity for malondialdehyde because other aldehydes and lipid oxidation products can also react with TBA, potentially contributing to the measured absorbance. In addition, matrix components present in certain meat products interfere with the determination and affect the accuracy of the results [105].

In this regard, separation techniques such as HPLC and GC are widely used for the determination of different secondary lipid oxidation products because of their versatility, separation capabilities, sensitivity, and potential for the simultaneous analysis of multiple compounds [106,107]. These techniques can be coupled with several detection systems, including ultraviolet (UV) and fluorescence detectors and mass spectrometry (MS) for HPLC, as well as flame ionization detection (FID) and MS for GC [108]. The selection of the separation and detection system depends on the physicochemical properties, volatility, stability, and polarity of the target compounds.

In addition to chromatographic separation, appropriate sample preparation and cleanup procedures are often required before analysis. These procedures facilitate the removal of interfering matrix components and, when necessary, the preconcentration of the target analytes, thereby improving analytical sensitivity and reliability [109]. Conventional sample preparation approaches include liquid–liquid extraction (LLE) and solid-phase extraction (SPE). More recently, several microextraction techniques have been developed as alternatives to conventional extraction procedures, offering reduced solvent consumption, lower sample volumes, and simplified sample preparation [110]. Consequently, a variety of analytical methodologies combining chromatographic separation, appropriate detection systems, and optimized sample preparation procedures have been developed for the determination of major lipid oxidation products, providing improved selectivity, sensitivity, precision, and accuracy (Figure 7).

Jin et al. (2021) [111] used GC-MS to identify the main volatile compounds associated with chicken meat aroma, detecting 44 compounds, including aldehydes, alcohols, and alkanes. Among these, hexanal and 1-octen-3-ol were the most abundant; these compounds are described as primary products of lipid degradation. In another related study, headspace solid-phase microextraction (HS-SPME) coupled with GC-MS was employed to evaluate volatile compounds associated with pork degradation. Initially, 47 compounds were detected, including 9 alcohols, 5 ketones, 2 aldehydes, 13 esters, 4 hydrocarbons, and 14 additional compounds. As degradation progressed, 66 compounds were detected, comprising 18 alcohols, 5 ketones, 6 aldehydes, 11 esters, 16 hydrocarbons and heterocyclic compounds, and 10 additional compounds. Ketones and aldehydes as well as octane, ethyl acetate, 1-pentanol, 1-hexanol, and 1-octen-3-ol which are primarily associated with meat lipid degradation products showed the greatest variation in concentration levels, while other compounds were linked to degradation sources such as carbohydrates and proteins. Furthermore, although these volatile compounds can provide valuable information regarding meat spoilage, not all are specific products of lipid oxidation, as some may also originate from microbial metabolism or the degradation of other food components [112].

Similar analyses have been performed in other meat species, including duck [113], turkey [114], pork [115], lamb [116], and beef [117], with SPME being one of the most frequently used sample preparation techniques when coupled with GC-MS [118]. Among the compounds that stand out most when evaluating lipid oxidation are: hexanal, octanal, nonanal, (E,E)-2,4-decadienal, 3-methyl-1-butanol, 4-hydroxy-2,5-dimethyl-3-(2H)-furanone, 1-penten-3-one, 2-pentylfuran, and methyl butyrate. Compounds such as methional, methanethiol, 2-furfurylthiol, 2-methyl-3-furanthiol, 3-mercapto-2-pentanone, and N-morpholinomethyl-isopropyl-sulfur are also described in the context of degradation processes; however, these are primarily attributed to proteins [118,119].

On the other hand, HPLC-based methods have been applied for the determination of specific lipid oxidation products and related groups of compounds. Compared to conventional spectrophotometric assays based on TBA, HPLC can improve selectivity in the determination of malondialdehyde by separating the compound of interest from other TBA reactive compounds. These methods typically involve separation on a reversed-phase C18 column, followed by UV-Vis detection commonly at around 530 nm or fluorescence detection (excitation = 530 nm/emission = 550 nm), depending on the analytical procedure [120].

Currently, HPLC-based methods have been increasingly applied to the analysis of aldehydes generated during lipid oxidation. Since many aldehydes lack a functional group suitable for detection by UV, fluorescence, or mass spectrometry, derivatization is necessary to introduce detectable functional groups such as chromophores, fluorophores, or ionizable groups [69]. Among the derivatization reagents used for this purpose, 2,4-dinitrophenylhydrazine (DNPH) which forms hydrazones is primarily employed for the determination of aldehydes and ketones. Although multiple compounds react with DNPH, coupling the reaction with chromatographic techniques imparts selectivity to the methodology, enabling the specific determination of aldehydes associated with lipid oxidation [70].

For example, the determination of 4-hydroxy-2-nonenal in grass carp, silver carp, bighead carp, common carp, crucian carp, and Wuchang bream, using derivatization with 2,4-dinitrophenylhydrazine and HPLC-UV quantification at 378 nm, limit of detection between 0.029 and 0.176 μmol kg^−1^ were obtained [121]. Similar strategy was developed for the simultaneous determination of 16 aldehydes (acrolein, propanal, crotonaldehyde, butanal, pentanal, trans-2-pentenal, hexanal, trans-2-hexenal, heptanal, trans, trans-2,4-heptadienal, octanal, trans, trans-2,4-octadienal, nonanal, trans, trans-2,4-decadienal, 4-hydroxy-2-hexenal, and 4-hydroxy-2-nonenal) and 8 ketones (acetone, 2-butanone, 2-pentanone, 2-hexanone, 5-hydroxy-2-pentanone, 2-octanone, 2-nonanone, and 2-decanone) in oysters. Carbonyl compounds were derivatized using 2,4-dinitrophenylhydrazine and analyzed by HPLC-MS, obtaining LODs in the interval of 0.2–20 µg L^−1^ [122].

Table 1 summarizes the main analytical techniques for determination of secondary lipid oxidation products in meat and meat products. In general, chromatographic methods offer greater selectivity than conventional spectrophotometric assays and allow for the determination of specific oxidation products, either individually or simultaneously. GC-MS, especially when combined with SPME or HS-SPME is particularly useful for characterizing the profile of volatile oxidation products, although not all the compounds detected can be attributed to lipids. Meanwhile, HPLC coupled with UV, fluorescence, or MS detection is suitable for determining oxidation products with low thermal stability, thereby enabling better identification of secondary oxidation compounds. Consequently, the complementary use of these techniques can provide a more comprehensive assessment of lipid oxidation in meat and meat products.

Other degradation products of interest are cholesterol oxidation products, particularly oxysterols. These compounds can be determined using both GC and HPLC techniques [127]. However, GC analysis generally requires a saponification step followed by derivatization with a silylation reagent to improve the volatility and chromatographic behavior of the analytes. Czerwonka et al. (2024) [128] developed a method for the simultaneous determination of squalene, cholesterol, 7-ketocholesterol, 7α-hydroxycholesterol, 7β-hydroxycholesterol, 25-hydroxycholesterol, 5,6α-epoxycholesterol, 5,6β-epoxycholesterol, and cholestanetriol in chicken breast, pork, salmon, and cod. The analytes were derivatized in pyridine using a silylation mixture consisting of N,O-bis(trimethylsilyl)trifluoroacetamide containing 1% trimethylchlorosilane, and the resulting derivatives were analyzed by GC-MS.

The determination of oxysterol content in food is useful for estimating dietary exposure to these compounds and for supporting food safety and risk assessment. Oxysterols have also been determined in meat products such as kabanos, bacon, salami, and pork. In this case, the samples were saponified with potassium methoxide, followed by derivatization of the analytes using anhydrous pyridine and a silylation reagent containing 1% trimethylchlorosilane. Cholesterol and oxysterols were subsequently analyzed by GC-MS, revealing the presence of 7α-hydroxycholesterol, 7β-hydroxycholesterol, cholestanetriol, and 7-ketocholesterol. These findings highlight the importance of monitoring sterol oxidation products in meat and meat products because of the potential health risks associated with dietary exposure to oxysterols [87]. Table 2 summarizes selected methodologies reported for the determination of oxysterols in different types of meat and meat products.

Lipid oxidation is a complex process in meat and meat products that generates a wide range of compounds capable of negatively affecting food quality and safety; some of these pose potential risks to human health. However, current regulatory frameworks offer limited specific guidance regarding the presence and permissible levels of various lipid oxidation products in meat and meat products. Consequently, there is a need for methods that enable the proper monitoring of these compounds and the acquisition of reliable analytical data on their presence and concentration in different meat matrices. In this regard, compounds such as ketones, oxysterols and saturated and unsaturated aldehydes including formaldehyde, acetaldehyde, acrolein, and crotonaldehyde require special attention due to their potential toxicological relevance. Chromatographic methods serve as valuable analytical tools for the identification, quantification, and monitoring of these compounds in meat and meat products, owing to their versatility and multi-analyte capability, as well as their adequate sensitivity, precision, and accuracy. Therefore, determining lipid oxidation products in meat and meat products using chromatographic methods can provide relevant information for exposure assessment. Furthermore, given their significance, regulatory criteria and permissible limits should be established for specific oxidation products in meat.

## 8. Conclusions

Lipid oxidation is a primary process affecting the quality of meat and meat products, as it can cause undesirable changes in color, texture, aroma, and flavor, while also contributing to the loss of certain nutritional components. Consequently, it is essential to determine lipid oxidation markers that allow for the assessment of the degree of oxidative deterioration and the formation of oxidation products that pose potential health risks.

In this context, chromatographic techniques have proven to be fundamental tools for identifying and quantifying primary and secondary lipid oxidation products, particularly due to their ability to provide specific information on various compounds. Although widely used analytical methods exist for certain oxidation indicators, further standardization is required to improve result reliability and selectivity. Moreover, the complexity of meat matrices and the diversity of oxidation products continue to pose a challenge for developing determination strategies with greater selectivity and sensitivity, capable of enabling more comprehensive characterization.

Furthermore, despite existing information linking certain oxidation compounds to the development of specific diseases, appropriate toxicological studies are needed to establish permissible exposure limits for lipid oxidation products found in meat. In this regard, regulatory standards focused on lipid oxidation products, which represent a risk to health as well as the quality of meat and meat products, will be implemented.

## Figures and Tables

**Figure 1:**
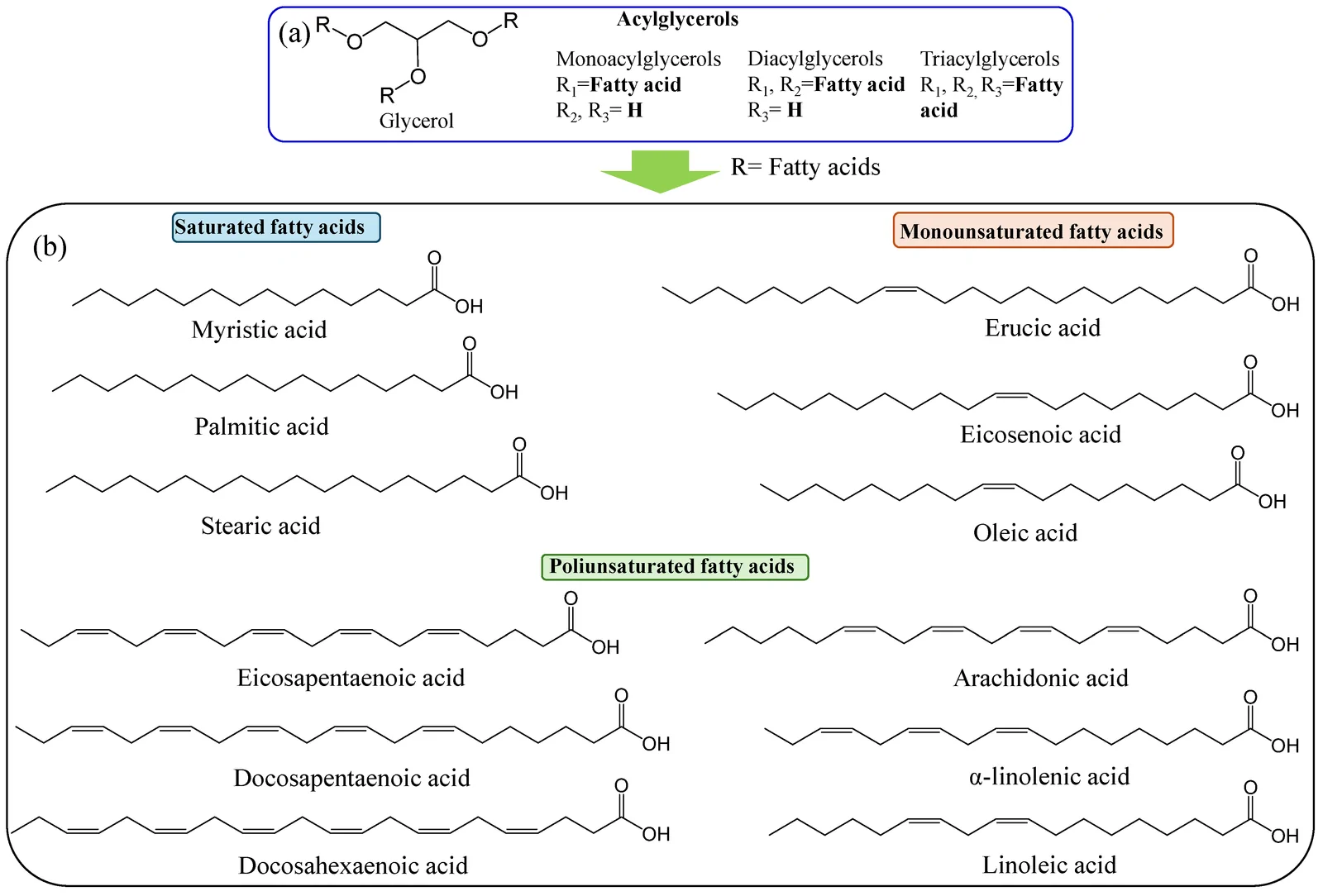
(**a**) Acylglycerols; (**b**) main fatty acids.

**Figure 2:**
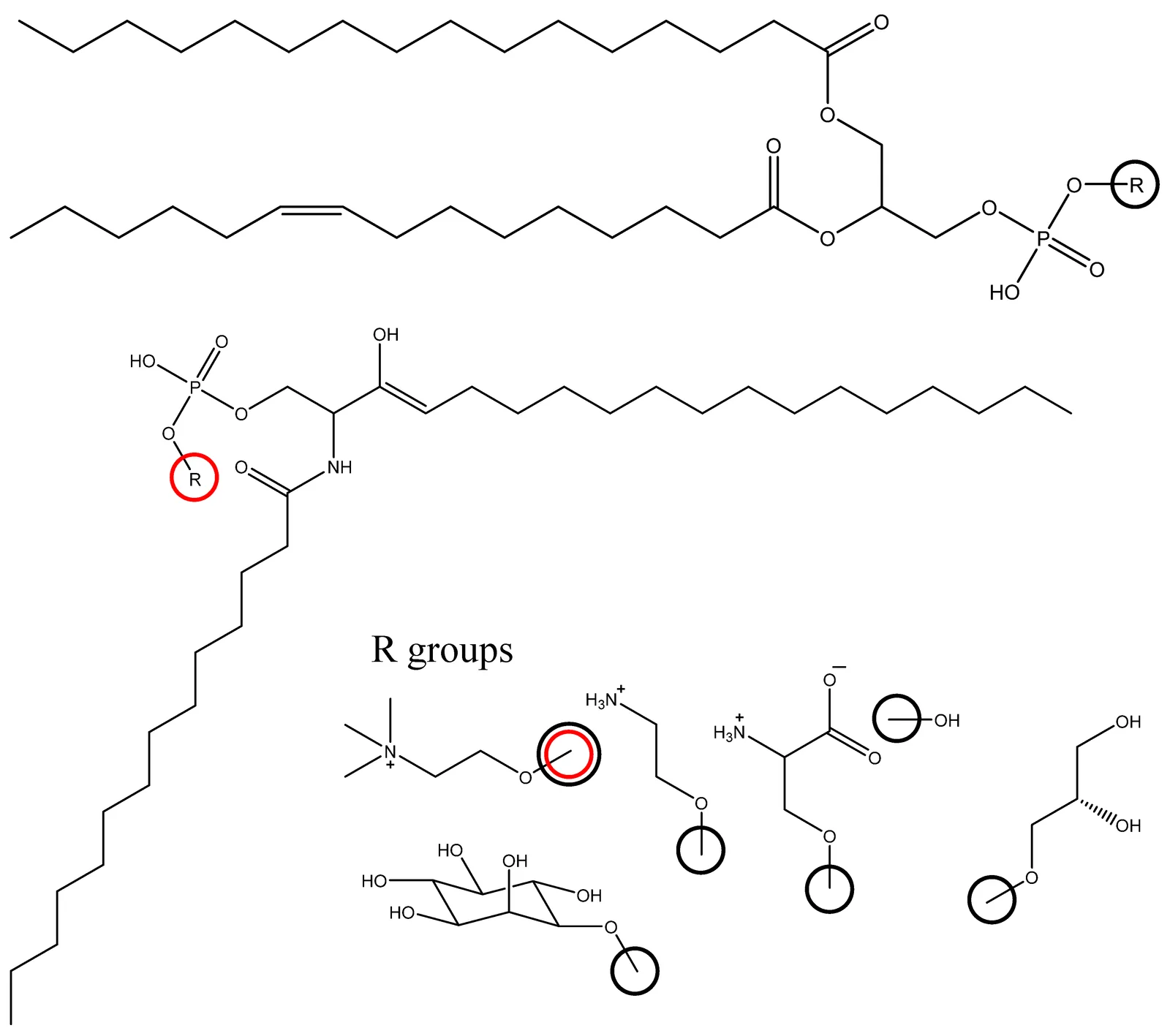
Basic structure of glycerophospholipids and sphingophospholipids as well as their main functional groups.

**Figure 3:**
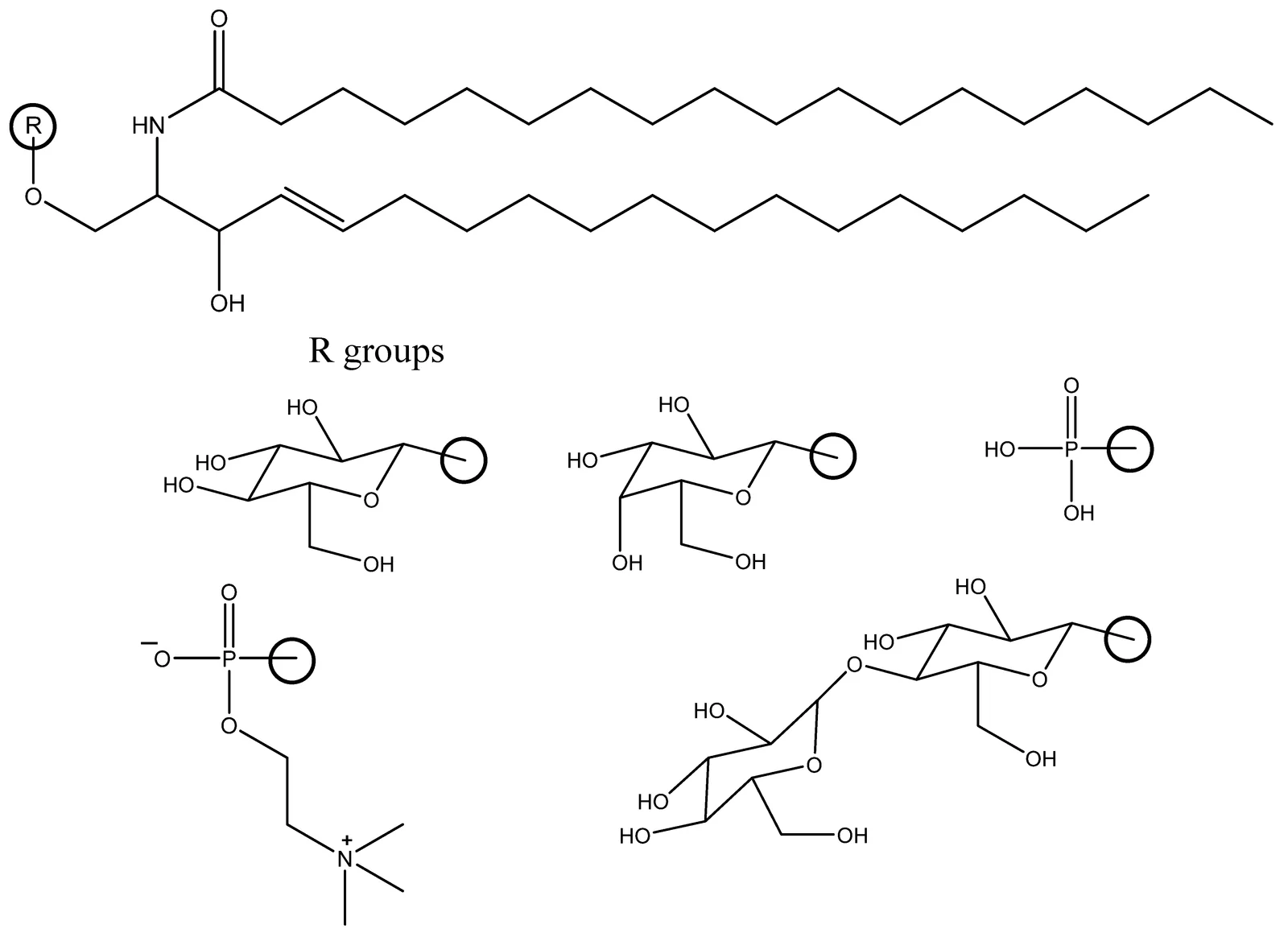
Basic structure of sphingolipids and their main functional groups.

**Figure 4:**
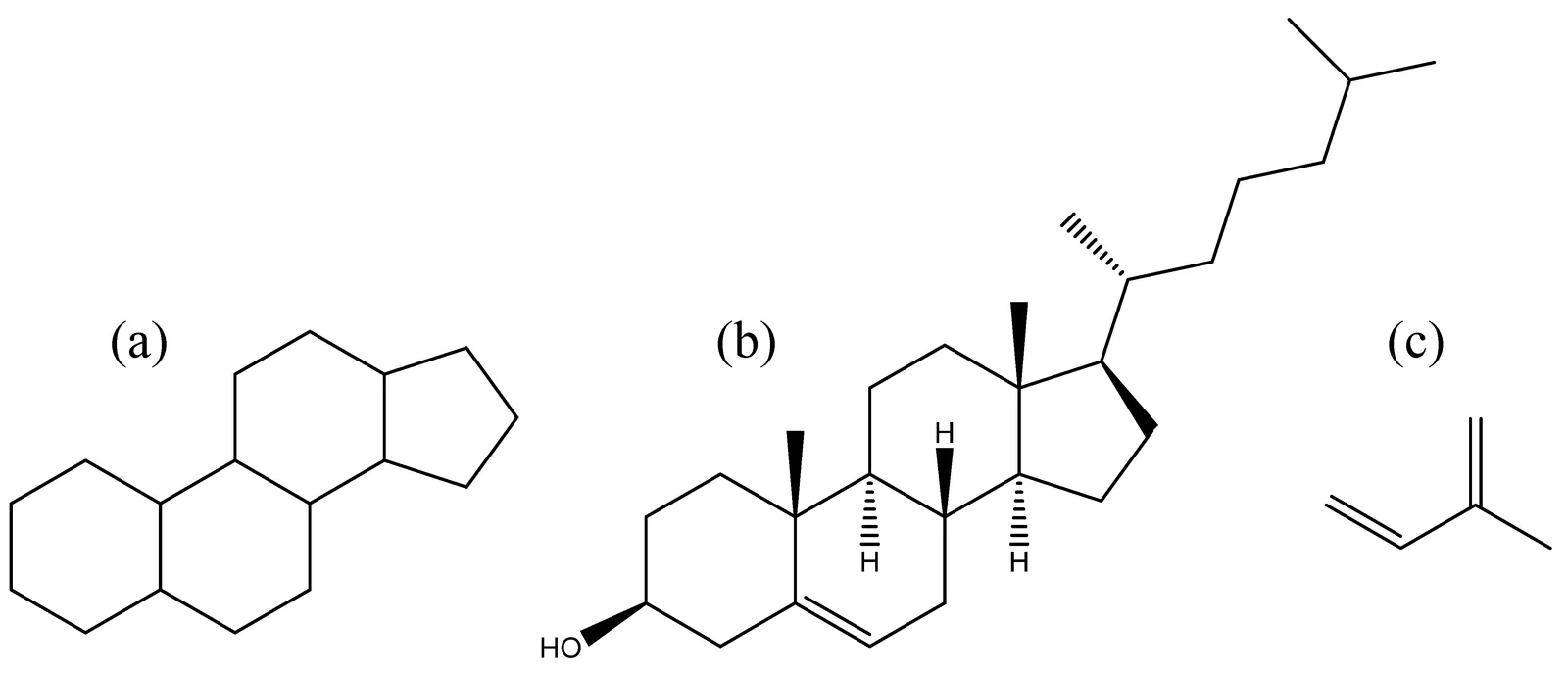
(**a**) Cyclopentanoperhydrophenanthrene, (**b**) cholesterol, and (**c**) isoprene.

**Figure 5:**
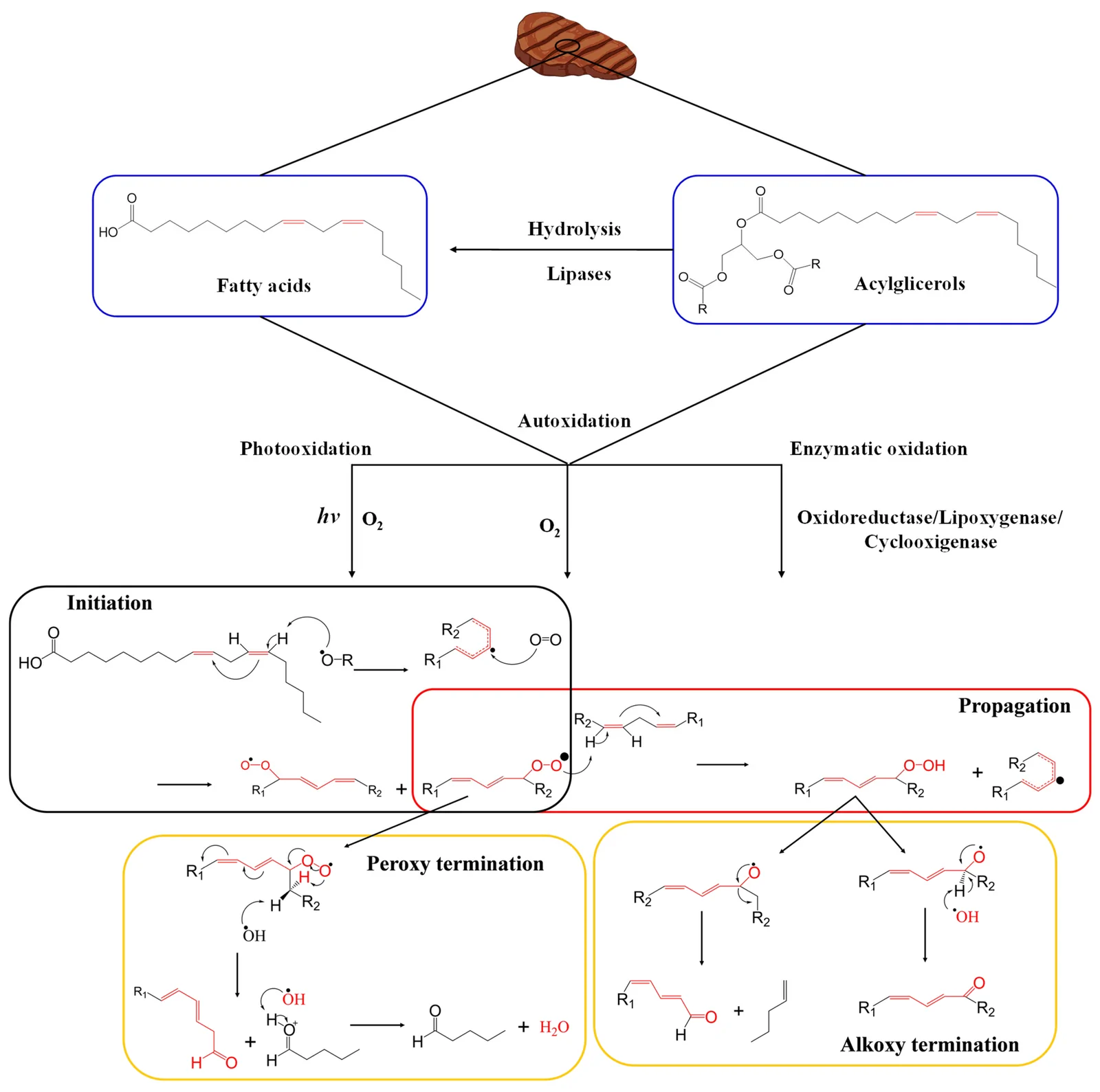
Main lipid oxidation pathways in meat and meat products.

**Figure 6:**
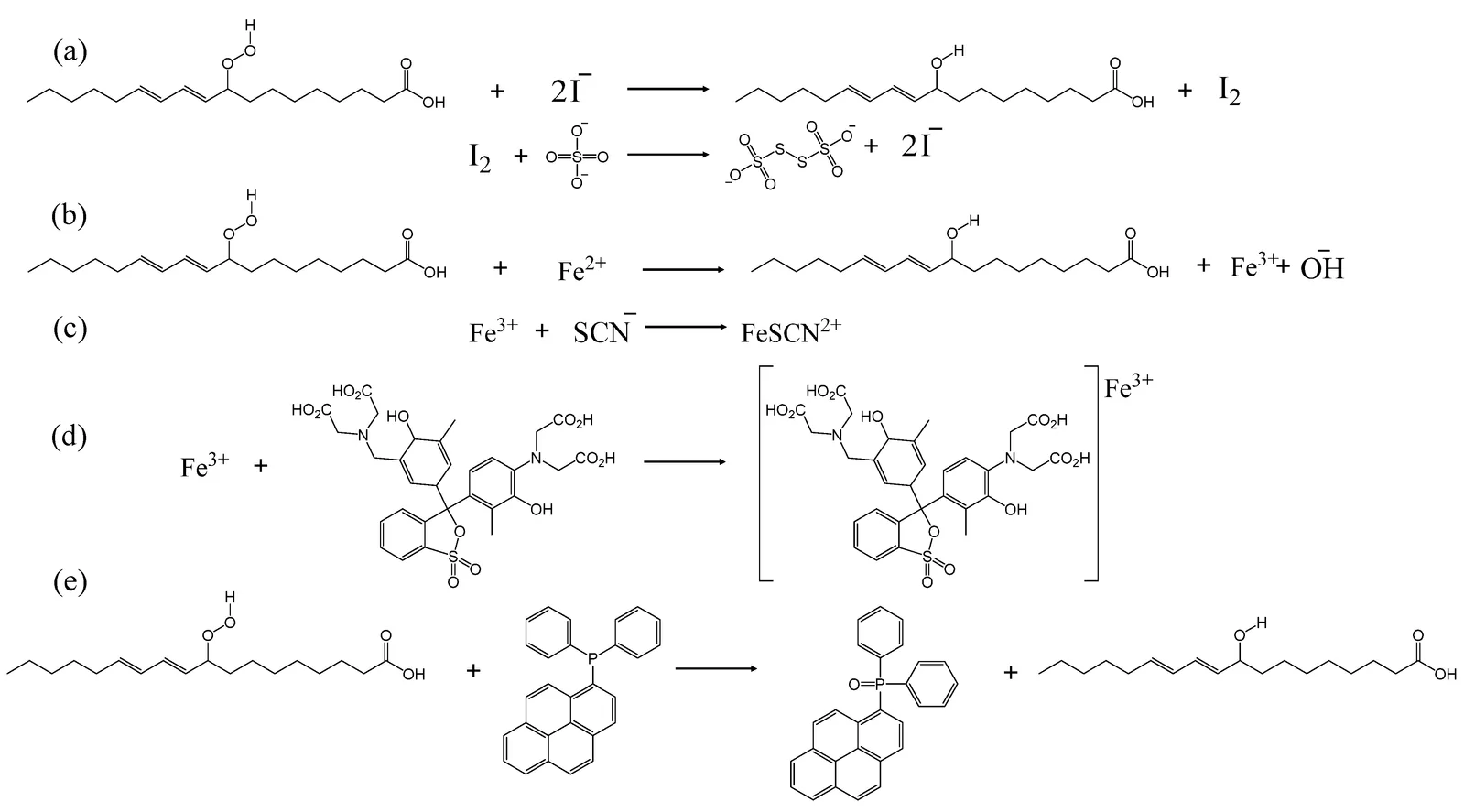
Reactions involved in the determination of lipid hydroperoxides: (**a**) iodometric titration reactions, (**b**) ferrous ion oxidation reaction, (**c**) formation of Fe (III)-thiosulfate complex, (**d**) formation of Fe (III)-Xylenol Orange complex, and (**e**) reaction with diphenyl-pyrenylphosphine.

**Figure 7:**
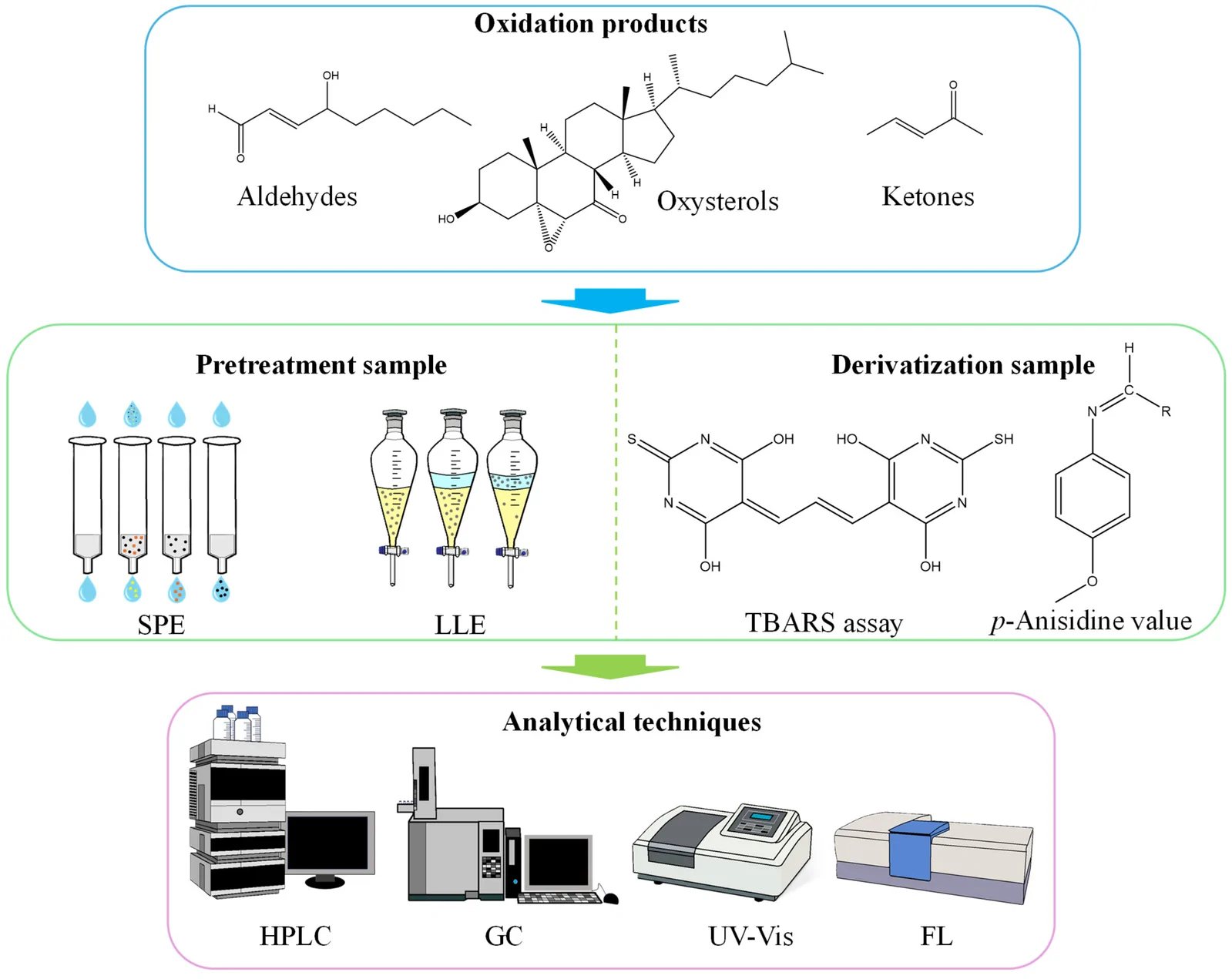
Procedure used for the analysis of lipid oxidation products in meat and meat products. SPE; solid-phase extraction, LLE: liquid–liquid extraction, TBARS; thiobarbituric acid reactive substances; HPLC; high-performance liquid chromatography, UV-Vis; ultraviolet-visible, FL; fluorescent.

**Table 1:** Main methods for the determination of lipid oxidation products in meat.

Oxidation Compounds	Meat Sample	Technique for Determination	Method Extraction	Derivatization Agent	LODs	Ref.
Malondialdehyde	Raw meat (pork, chicken, beef and lamb). Processed meat (chorizo, salchichon, frankfurter sausage, cooked ham and beef burger)	HPLC-FLD	Solid–liquid Extraction	2-thiobarbituric acid	0.91 µmol L^−1^	[120]
Crotonaldehyde, butanal, pentanal, trans-2-hexenal, hexanal, trans, trans-2,4-heptadienal, heptanal, trans, trans-2,4-octadienal, octanal, nonanal, and trans, trans-2,4-decadienal	Pork, beef, mutton, chicken, chicken wings, and fish	HPLC-MS	SPE	2,4-dinitrophenylhydrazine	0.010–0.621 µg kg^−1^	[106]
16 aldehydes (acrolein, propanal, crotonaldehyde, butanal, pentanal, trans-2-pentenal, hexanal, trans-2-hexenal, heptanal, trans, trans-2,4-heptadienal, octanal, trans, trans-2,4-octadienal, nonanal, trans, trans-2,4-decadienal, 4-hydroxy-2-hexenal and 4-hydroxy-2-nonenal) 8 ketones (acetone- 2-butanone- 2-pentanone, 2-hexanone, 5-hydroxy-2-pentanone, 2-octanone, 2-nonanone, 2-decanone)	Oyster	HPLC-MS	Solid-liquid extraction	2,4-dinitrophenylhydrazine	0.2–20 µg L^−1^	[122]
Duck (16 aldehydes, 9 alcohols, 14 alkanes, 3 ketones, 7 esters, and 7 other compounds). Chicken (13 aldehydes, 6 alcohols, 17 alkanes, 3 ketones, 12 esters, and 3 other compounds). Beef (14 aldehydes, 7 alcohols, 13 alkanes, 3 ketones, 4 esters, and 3 other compounds). Mutton (15 aldehydes, 6 alcohols, 11 alkanes, 3 ketones, 5 esters, and 3 other compounds). Pork, including 11 aldehydes, 5 alcohols, 10 alkanes, 2 ketones, 5 esters, and another compound.	Chicken, duck, pork, beef, and mutton	GC-MS	HS-SPME		N.R	[107]
4-hydroxy-2-nonenal	Grass carp, silver carp, bighead carp, common carp, crucian carp, and Wuchang bream.	HPLC-UV	Solid–liquid extraction	2,4-dinitrophenylhydrazine	0.029–0.176 μmol kg^−1^	[121]
Propanal, pentanal, hexanal, heptanal octanal, and nonanal	Turkey	GC-FID	HS-SPME		0.9–11.6 μg kg^−1^	[123]
4-hydroxy-2-hexenal and 4-hydroxy-2-nonenal	Bluefin tuna, blue mackerel, red sea bream, yellowtail, beef, pork and chicken	HPLC-FLD		1,3-cyclohexanedione	27–30 μmol L^−1^	[124]
2-Methylbutanal, 3-Methylbutanal, pentanal, hexanal, heptanal, octanal, nonanal, decanal, dodecanal, (E)-2-heptenal, (E)-2-octenal, (E)-2-nonenal, (E)-2-decenal, (E,E)-2,4-decadienal, benzaldehyde, phenylacetaldehyde, 2-heptanone, acetoin, and 2-methylpentanal	Roasted lamb	GC-MS	HS-SPME	O-2,3,4,5,6-(pentafluorobenzyl) hydroxylamine hydrochloride	0.01–100.00 µg L^−1^	[125]
Formaldehyde, acetaldehyde, pentanal, hexanal, heptanal, octanal, nonaldehyde, and decanal	Pork	HPLC-FLD		2-(12-benzo[b]acridin-5(12H)-yl)-acetohydrazide	0.30–0.45 μg kg^−1^	[126]

GC; gas chromatography, HPLC; high-performance liquid chromatography; MS; mass spectrometry, FID; flame ion detection, SPE; solid-phase extraction, FLD; fluorescence detector, UV; ultraviolet, HS-SPME; headspace-solid phase microextraction.

**Table 2:** Main methods for the determination of oxysterols in meat.

Oxidation Compounds	Meat Sample	Technique for Determination	Method Extraction	Derivatization Agent	LODs	Ref.
5,6α-epoxycholesterol, 5,6β-epoxycholesterol, 7-keto cholesterol, 25-hydroxycholesterol, 5α-cholestane-3β,-5α, 6β-triol 7α-hydroxy cholesterol and 7β-hydroxy cholesterol	chicken cutlet, pork cutlet, sausage, sauryfish, boiled pork and smoked chicken	GC-MS	QuEChERS	N,O-bis(trimethylsilyl)acetamide, trimethyl chlorosilane and N-trimethylsilylimidazole (3:2:3 *w*/*w*/*w*)	2–400 µg kg ^−1^	[129]
5α,6α-epoxycholestanol, 5β,6β-epoxycholestanol, 3,5,6-triolcholesterol, 7-ketocholesterol, 7α-hydroxycholesterol, 7β-hydroxycholesterol, 25-hydroxycholesterol and cholesterol	Oyster	HPLC-MS	LLE	4-(dimethylamino)phenyl isocyanate	0.010–0.621 µg kg ^−1^	[130]
Squalene, cholesterol, 7-ketocholesterol, 7α-hydroxycholesterol, 7β-hydroxycholesterol, 25-hydroxycholesterol, 5,6α-epoxycholesterol, 5,6β-epoxycholesterol, cholestanetriol	Chicken breast meat, pork chops, salmon, and cod	GC-MS	LLE	N,O-Bis(trimethylsilyl)trifluoroacetamide with 1% of trimethylchlorosilane	0.01 mg L^−1^	[128]
20α-hydroxycholesterol, 7-ketocholesterol, 7β-hydroxycholesterol, 22R-hydroxycholesterol and 25-hydroxycholesterol	Cypriot Meat	HPLC-DAD	SPE		0.99–1.28 mg L^−1^	[131]
7*β*-hydroxycholesterol, cholesterol 5*β*,6*β*-epoxide, cholesterol 5*α*,6*α*-epoxide, cholestane-3*β*,5*α*,6*β*-triol, 7-ketocholesterol, 25-Hydroxycholesterol	Beef, cooked ham, Serrano ham and smoked salmon	GC-FID	SPE	1,1,1,3,3,3-hexamethyldisilazane/trimethylchlorosilane	12–63	[132]
Cholestane-5α-6α-epoxide, cholestane-3β-5α-6β-triol, 25-hydroxycholesterol and 5-cholesten-3β-ol-7-one	Iberian dry-cured ham	GC-FID	LLE, SPE	Pyridine: Chlorotrimethylsilane: hexamethyldisilazane (9:3:1, *v*/*v*/*v*)	0.03 mg kg^−1^	[133]
7α-hydroxycholesterol, 7β-hydroxycholesterol, cholesterol triol, cholesterol and 7-ketocholesterol	Kabanos, bacon Salami and Pork	GC-MS	LLE	N,O-bis(trimethylsilyl)trifluoroacetamide with 1% of trimethylchlorosilane	0.2–0.5 mg L^−1^	[87]

GC-MS; gas chromatography-mass spectrometry, HPLC; high-performance liquid chromatography, FID; flame ion detection, SPE; solid-phase extraction, LLE; liquid–liquid extraction, DAD; Diode Array Detector, QuEChERS; Quick, Easy, Cheap, Effective, Rugged and Safe.

## Data Availability

No new data were created or analyzed in this study. Data sharing is not applicable to this article.
